# 

**Published:** 1896-12-26

**Authors:** 


### EARLY EDITION.

**Notices of Births, Deaths, and Marriages** are inserted in this column at a charge of 2s. 6d. for an announcement not exceeding four lines.

### NOTICE TO CORRESPONDENTS.

All MS., Letters, Books for Review, and other matters intended for the Editor should be addressed The Editor, "The Hospital," The Hospital Building, 28 & 29, Southampton Street, Strand, W.C.

The Editor cannot undertake to return rejected MS., even when accompanied by stamped directed envelope.

**Notice to Advertisers and Subscribers.**

All Advertisements, Orders for Copies of The Hospital, and Business Communications should be addressed to **THE MANAGER, (Not to The Editor),** The Hospital, The Hospital Building, 28 & 29, Southampton Street, Strand, London, W.C.

The Cost of Subscription, post free, is as follows:—Per week, 2½d.; per quarter, 2s. 8d.; per half-year, 5s. 3d.; per annum, 10s. 6d. Foreign:—Per annum, 15s. 2d.; payable, in all cases, in advance.

**NOTICE.—Vol. XX. of "The Hospital" (April to September, 1896), in handsome cloth binding, is now on sale at the Office of this Journal, price 7s. 6d. Cases for binding the Numbers contained in this Volume 1s. 6d. Index to Vol. XX., 2½d., post free. The Monthly Part for January will be ready on January 1st. Price 6d., by post 8d.**

### Scraps and Gleanings.

The first patient has been received into the Oban Cottage Hospital.

Contracts have been entered into for the erection of the new workhouse hospital for Halifax.

It is proposed to establish a convalescent home in connection with the Leeds Workpeople's Hospital Fund.

A memorial bed in memory of the late Sir George Porter, Bart., has been opened at the Meath Hospital, Dublin.

The Liverpool Hospital Sunday collections for 1896 have amounted to £6,334, against £6,156 in 1895 and £6,265 in 1894.

The Corporation of Edinburgh are about to erect a new hospital for the treatment of infectious diseases at Colinton Mains, Midlothian.

The Coventry Hospital Sunday Fund has raised £1,336 this year at an expense of £25. This amount is the largest sum collected so far by the fund.

The corner stones of the two new wards which are to provide the much-needed increased accommodation at the Blackpool Hospital were laid last month.

As an addition to the Midland Counties Home for Incurables at Leamington, a new home for ladies and gentlemen is to be erected, with accommodation for 60 persons.

The Driffield Cottage Hospital during the year just closed only received £271, against an expenditure of £326, leaving a balance due to the treasurer on the year's working of £55.

The patients treated at the Temple Street Children's Hospital, Dublin, during the year ended September 1st, 1896, numbered 676, or 126 more than last year. Over 6,000 cases attended as out-patients.

The Croydon General Hospital treated 836 in-patients in 1895-6, or 134 more cases than in 1894-5, and 273 more than in 1893-4. The income for 1895-6 amounted to £4,093, and the expenditure on maintenance to £4,260.

The expenditure at the Glasgow Maternity Hospital during the year just ended was £2,030, almost identically the same as that for the previous year. Four hundred and eighty-two in and 2,138 out patients were attended.

The report of the Ballymena Cottage Hospital for the year ended October 31st, 1896, just issued, is satisfactory. The receipts amounted to £452, and the expenditure to £418, leaving a balance in hand of £34. One hundred and ten in and 18 out patients were treated.

The accounts of the Stranraer Cottage Hospital for the year ended September 30th, 1896, show a balance in favour of the hospital of over £37 on the year's working, the income having been £274 and the expenditure £237. The reserve fund now amounts to £230.

The year ended September 30th, 1896, has been one of increased work at the Derbyshire Royal Infirmary. The number of patients treated has increased all round—in-patients from 1,307 in 1894-5 to 1,418 in 1895-6; out-patients from 5,644 to 6,333; and casualties from 2,595 to 2,798—and the daily average number of beds occupied jumped, in consequence, from 110 to 115.

The total population of the two asylums of the Central Provinces of India during 1895 was 381, or five in excess of that of 1894. The percentage of patients cured to total population was 6·30, against 5·32 in 1894. The total cost of the asylums was Rs.25,176; and the average annual cost per lunatic was at Nagpur Asylum, Rs.64 5a. 10p.; and at Jubbulpore Asylum, Rs.61 13a.

During the year ended August 31st, 1896, 2,015 in-patients were treated at the Belfast Royal Hospital, 386 at the convalescent home connected therewith, 36 in the consumptive department, and 93 in the Children's Hospital. Twenty-three thousand four hundred and four out-patients were also treated. In 1888 the average number of days each case was resident was 16½, while in 1895-6 it had risen to 25¼.

### The Student of Medicine.

### APPOINTMENTS.

Mr. H. Athill-Cruittwell, appointed Medical Officer for the Hollaton District of the Uppingham Union. F. A. Baldwin, M.R.C.S., L.R.C.P., L.S.A., appointed Public Vaccinator to the Fifth Vaccination District of the St. Saviour's Union. Mr. R. Brown, appointed Medical Officer for the No. 8 District of the Croydon Union. B. I. D'Olier, B.A.Dubl., M.B., appointed Medical Officer for the Arundel District of the East Preston Union. C. H. East, M.D., B.S.Durh., M.R.C.S., appointed Honorary Surgeon to the Malvern Rural Hospital. Mr. F. V. Elkington, appointed Medical Officer for the Burton Bassett District of the Southam Union. Mr. T. Gibson, jun., appointed Medical Officer of the Workhouse of the Sheffield Union. J. T. M. Giffen, L.R.C.P., L.R.C.S.Edin., L.F.P.S.Glasg., appointed Medical Officer of the Workhouse of the Tarvin Union. D. Hunter, M.A., M.B., B.C.Cantab., L.S.A., appointed Pathologist to the County Asylum, Whittingham, Lancashire. J. W. H. Jellett, M.D.Dubl., appointed Medical Officer of Health to the Heavitree Urban District Council. Gertrude Keith, L.R.C.P. and S. Edin., appointed Senior House Surgeon to the New Hospital for Women, Euston Road, N.W. T. H. Kellock, F.R.C.S.Eng., &c., appointed Surgical Registrar to the Middlesex Hospital. J. Lewis, M.R.C.S.Eng., L.S.A., appointed Medical Officer of Health to the Aberayron Rural District Council. E. I. Maclean, M.D., M.R.C.P., appointed Physician to Out-patients at the Chelsea Hospital Women. E. A. Malcolmson, L.R.C.P., L.R.C.S.I., appointed Medical Officer to the Cavan Dispensary District. J. H. Murray, M.B., C.M.Edin., appointed Medical Officer of Health to the Stirling Parish Council. H. W. Newton, M.R.C.S., L.R.C.P., D.P.H., appointed Medical Officer to the 1st District of the Chelmsford Union, and Surgeon to the Essex County Police. C. T. Parsons, M.D.Lond., appointed Assistant Medical Officer to the Fulham Union Infirmary. R. J. Probyn-Williams, M.D.Durh., L.R.C.P.Lond., M.R.C.S.Eng., appointed Assistant Instructor in Anæsthetics at the London Hospital. G. A. Robinson, M.R.C.S.Eng., L.R.C.P.Lond., L.D.S.Eng., elected Resident Surgeon to the Nottingham General Dispensary. A. Roxburgh, M.B., C.M., appointed Dispensary Surgeon to the Bellahouston Dispensary. J. A. Smith, M.D.Lond., appointed Honorary Medical Officer of the Kilburn Dispensary, and Medical Officer for the No. 1 District of the Willesden Union. W. D. Spurrell, M.R.C.S.Eng., L.R.C.P.Lond., appointed Medical Officer for the Aldborough District of the Erpingham Union. M. Thorne, L.S.A.Lond., M.B.Brux., appointed Junior House Surgeon to the New Hospital for Women, Euston Road, N.W. W. R. Turnham, M.B., B.S.Durh., appointed Second Assistant Medical Officer to the Lunatic Asylum at Fishponds. A. E. L. Wear, M.D., B.S.Durh., appointed Anæsthetist to the Hospital for Women and Children, Leeds. F. H. Westmacott, F.R.C.S.Eng., appointed Anæsthetist to the Manchester Hospital for Sick Children, Pendlebury. J. G. Wilson, M.A., M.B., C.M., appointed Dispensary Surgeon to the Victoria Infirmary, Glasgow. J. Jackson Clarke, M.B., Lond., F.R.C.S., Eng., has been appointed Assistant Surgeon to the City Orthopædic Hospital.

### Medical Appointment.

RESIDENT MEDICAL OFFICER WANTED for LIVERPOOL HAHNEMANN HOSPITAL and HOMŒOPATHIC DISPENSARIES, Hope Street. Must be a registered practitioner. Salary £80 per annum, with board, &c. Apply to the Secretary, Thos. Cooper, at the hospital. (746)

**Account Books for Institutions designed in accordance with the Uniform System of Accounts for Hospitals and Institutions.**
By HENRY C. BURDETT.

*Descriptive Price List, post free, on application.*

London: The Scientific Press (Limited), 28 & 29, Southampton Street, Strand, W.C.

